# Echocardiographic Assessment of Right Ventricular Function in Patients With Liver Cirrhosis

**DOI:** 10.7759/cureus.57410

**Published:** 2024-04-01

**Authors:** Augustine A Enenche, Anthony G Kweki, Henry O Aiwuyo, Anna Nevolina, Oluwasegun M Akinti, Jamal C Perry, Yonael Ayinalem, John O Osarenkhoe, Emmanuel Ukenenye, Charles O Poluyi, Solomon Danbauchi Sulei

**Affiliations:** 1 Internal Medicine, Jos University Teaching Hospital, Jos, NGA; 2 Internal Medicine/Cardiology, Dalhatu Araf Specialist Hospital, Lafia, NGA; 3 Internal Medicine/Cardiology, Colchester Hospital, East Suffolk and North Essex NHS Foundation Trust, Colchester, GBR; 4 Internal Medicine, Brookdale University Hospital Medical Center, Brooklyn, USA; 5 Medicine, Brookdale University Hospital Medical Center, Brooklyn, USA; 6 Medicine and Surgery, Igbinedion University Teaching Hospital, Benin City, NGA

**Keywords:** echocardiography, cardiovascular complications, liver cirrhosis, diastolic dysfunction, systolic dysfunction, right ventricle

## Abstract

Background: In patients with chronic liver disease, the common endpoint of its course is liver cirrhosis which is a cause of cardiovascular morbidity and mortality. These abnormalities in the cardiovascular system, especially the heart, can be detected by echocardiography. Identifying and acting on these abnormalities can have an impact on their management thereby reducing morbidity and mortality of patients with liver cirrhosis. The aim of this study was to determine the prevalence of right ventricular systolic and diastolic dysfunction in liver cirrhosis patients.

Methods and materials: A hospital-based cross-sectional study was conducted among adult patients of the gastroenterology unit (ward and clinic) diagnosed with liver cirrhosis. A total of 243 patients were recruited and 210 were evaluated for this study. This study was carried out over one year. Cardiology studies, including electrocardiography and echocardiography, were conducted on patients to assess right ventricular function.

Results: Among the participants, 44.8% had right ventricular hypertrophy and 3.8% had right ventricular dilatation. Using Tricuspid Annular Plane Systolic Excursion (TAPSE), 17.1% were found to have right ventricular systolic dysfunction and 51.4% had systolic dysfunction using FAC. Diastolic dysfunction was found in 61% of the participants and grade 2 diastolic dysfunction was the commonest.

Conclusion: From this study, a high prevalence of right ventricular systolic and diastolic dysfunction was recorded among patients with liver cirrhosis.

## Introduction

Cirrhosis of the liver is characterized as a progressive, diffuse fibrosing nodular disorder that destroys the overall normal hepatic architecture [1,2]. It is the common endpoint of chronic liver diseases regardless of the aetiology of liver disease. The topmost causes of liver cirrhosis include: alcohol, non-alcoholic steatohepatitis, and viral hepatitis [3]. Other less common causes of liver cirrhosis are autoimmune hepatitis, biliary cirrhosis, cardiac cirrhosis, hemochromatosis, Wilson's disease, cystic fibrosis, alpha 1 antitrypsin deficiency, and cryptogenic causes [4].

Liver cirrhosis is seen worldwide, however, with varying incidence and prevalence depending on the age groups, sex, ethnicity, and geographical locations [5,6]. Chronic liver cirrhosis constitutes about 46% of global diseases as well as 59% of global mortality with over 35 million people dying of chronic diseases yearly [5]. It is the fifth most common cause of mortality in Mexico and the United Kingdom [5,7]. Liver cirrhosis has been recorded as the 11th cause of death globally [8,9].

Cardiac abnormalities have been recorded in liver cirrhosis patients [10]. These cardiac abnormalities include left and right ventricular systolic and diastolic dysfunction, cirrhotic cardiomyopathy, pulmonary hypertension, and coronary artery disease [11-16]. Cirrhotic patients have been found to have biventricular dilatation and impaired biventricular systolic function [17]. It has been shown that right ventricular diastolic dysfunction is directly related to the degree of hepatosteatosis in non-alcoholic steatohepatitis [13].

In patients with liver cirrhosis, there is a depressed systolic response to stress and this is attributed to the down-regulation of beta-adrenergic receptors [18]. There is decreased content of G-protein, upregulation of cannabinoid 1-receptor, an increased inhibitory effect of cardio-depressive substances (heme oxygenase (HO), carbon monoxide (CO), nitric oxide synthase (NOS) induced nitric oxide (NO) release and tumour necrotic factor (TNF) [18]. All these tend to blunt the systolic response to stress in patients with liver cirrhosis [18]. It has also been implicated in renal failure in advanced disease [19]. Subtle systolic dysfunction at rest has been found more commonly using speckle-tracking echocardiography [18]. Eman et al. found lower fractional shortening among people with cirrhosis compared to non-cirrhotic patients [20]; however, the authors did not find significant changes in tricuspid annular plane systolic motion (TAPSE) among the two groups. Right ventricular global strain (RVGST) is highly negative and correlates positively with serum albumin [20]. Chen et al. have shown impairment in systolic strain among liver cirrhosis patients compared with controls despite a normal right ventricular fractional area of change (RVFAC) and TAPSE [17].

Moller et al. postulated that left ventricular diastolic dysfunction is due to cardiac stiffness following myocardial hypertrophy, fibrosis, and subendocardial oedema [18]. Sodium retention in liver cirrhosis due to the activation of the renin-angiotensin-aldosterone system (RAAS) partly explains the reason for diastolic dysfunction in patients with liver cirrhosis [18]. Diastolic dysfunction predicts death in patients with liver cirrhosis with a transjugular intrahepatic shunt (TIPS) and also correlates with hepatic decompensation [18]. Diastolic dysfunction in cirrhotic patients tends to improve following liver transplantation [18]. Several parameters have been used to assess the diastolic function of the left and right ventricles (RVs) in patients with liver cirrhosis. These include the ratio of the early and late diastolic filling (E/A), deceleration time (DT), isovolumic relaxation time (IVRT), and early diastolic mitral annulus velocity ratio (E/e') [18,20,21].

Studies assessing right ventricular function in liver cirrhosis in Nigeria are scarce, and one available study in Nigeria assessed left ventricular function [22].

Generally, an assessment of the RV is scarcely done. This study gives insight into right ventricular abnormality in patients with liver cirrhosis, thus reducing the dearth of knowledge about the cardiovascular complications of liver cirrhosis. 

## Materials and methods

A hospital-based cross-sectional analytical study was conducted among adults with liver cirrhosis at Jos University Teaching Hospital (JUTH), Jos, Nigeria. Participants were recruited from gastroenterology clinic and wards. The study was carried out within a 12-month period. The study was approved by the Human Research and Ethics Committee of Jos University Teaching Hospital (JUTH) (approval number: JUTH/DCS/IREC/127/XXX/2096).

Using Bennet Fisher's formula [23] and a prevalence of right ventricular hypertrophy among patients with liver cirrhosis reported to be 16% [24], an estimated sample size of 206 was obtained. A total of 243 participants were recruited; however, only 210 were analysed. A convenience sampling technique was used to choose the participants.

An interviewer-administered questionnaire was used to obtain information on demographic data, history suggestive of liver cirrhosis, and symptoms of cardiac dysfunction. Thereafter, patients had a detailed examination. Echocardiography was carried out on each respondent to assess right ventricular systolic function using TAPSE and RVFAC. TAPSE < 1.6 cm suggests longitudinal systolic dysfunction [21]. The RVFAC was calculated using (right ventricular end‐diastolic area (RVEDA)-right ventricular end‐systolic area (RVESA)/RVEDA multiplied by 100 with a lower reference value for normal RV systolic function of 35% [21].

The diastolic function of the RV was assessed by the following parameters: E/A, E/e', and DT. The diastolic function was graded as follows; E/A 0.8-2.1 was taken as normal, E/A <0.8 was taken as impaired relaxation, E/A 0.8-2.1 with E/e' >6 was regarded as pseudo filling, and E/A >2.1 with DT <120 ms was taken as restrictive filling [21].

Data were analysed using IBM SPSS Statistics for Windows, Version 23.0 (Released 2015; IBM Corp., Armonk, New York, United States). The data obtained were presented in tables and charts. Categorical variables were expressed as proportions and frequencies while continuous data as median, mean and standard deviation. Student t-test was used to compare the mean FibroScan score of patients with systolic dysfunction and those with normal systolic function. Analysis of variance (ANOVA) was used to compare the mean of FibroScans of patients with grade 1, 2, and 3 diastolic dysfunctions. A scatter plot was generated using Pearson correlation and was used to plot FibroScan score against systolic function using TAPSE and FAC.

## Results

Social demographic characteristics

**Figure 1:**
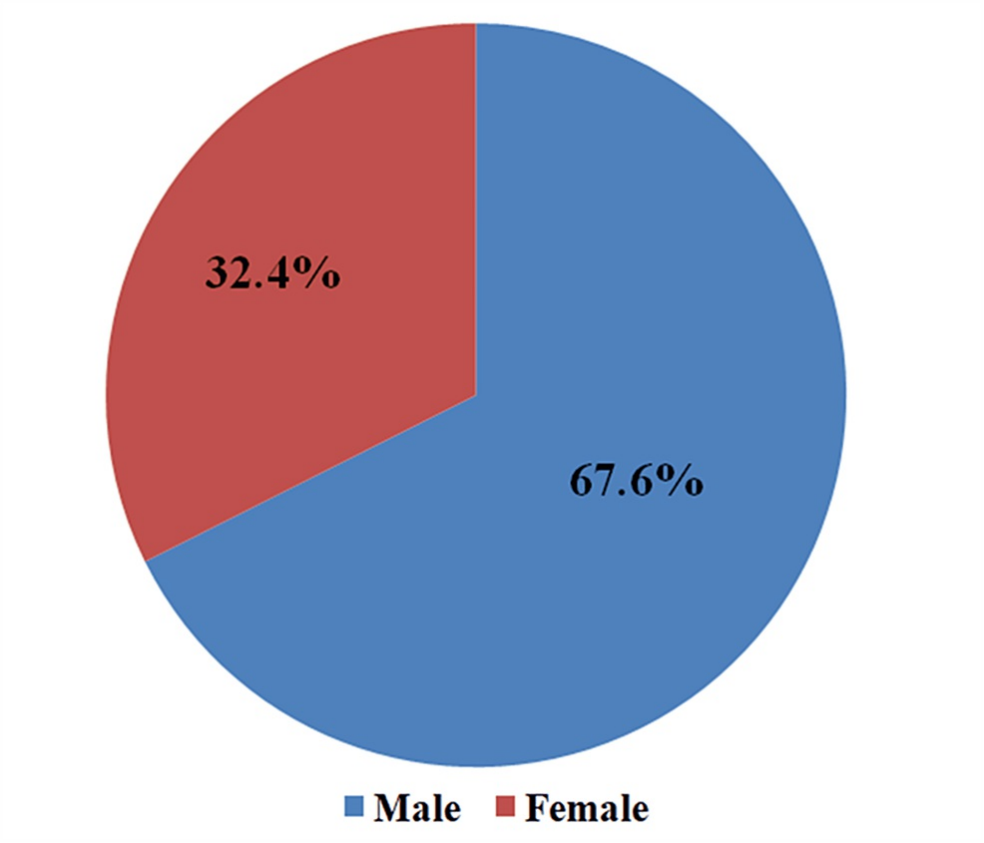
Gender distribution of study participants Data presented as Percentage (%)

As shown in Table 1 and Figure 1, the mean age of the participants was 47± 12.47 years. More than half of the participants were males (n=142; 67.6%). The participants were mostly married (n=174;82.9%) and six (2.9%) were widowed, Single participants were 28 (13.3%) and two (1.0%) were divorced. They were mostly urban dwellers (n=118; 56.2%) and rural dwellers were 92 (43.8%). Only 78 (37.1%) had secondary education, 60 (28.6%) had tertiary education, and 40 (19.0%) had primary education. Among the participants, four (1.9%) had informal education and 28 (13.3%) had no education. The most common occupations among the participants were farming and civil service with 60 (28.6%) each and trading was next accounting for 42 (20.0%) study participants.

**Table 1:** Demographic characteristics of patients Data presented as n and %, except for age, which was in mean±SD

Demographic characteristics	Frequency (n)	Percentage (%)
Age (years), mean ± SD	47±12.47	
Marital status		
Single	28	13.3
Divorced	2	1.0
Married	174	82.9
Widowed	6	2.9
Educational level		
None	28	13.3
Informal	4	1.9
Primary	40	19.0
Secondary	78	37.1
Tertiary	60	28.6
Occupation		
Student	4	1.9
Civil servant	60	28.6
Trader	42	20.0
Farmer	60	28.6
Housewife	8	3.8
Retiree	8	3.8
Artisan	12	5.7
Others	16	7.6
Residence		
Urban	118	56.2
Rural	92	43.8

Clinical characteristics

The most common clinical symptom was abdominal swelling among 120 (57.1%) participants; easy fatigability was seen in 96 (45.7%) and pedal swelling was seen in 80 (38.1%) (Table 2). Only four (1.9%) participants had orthopnoea and paroxysmal nocturnal dyspnoea each. Among the participants, 116 (55.2%) had hepatitis B virus infection and 70 (33.3%) had hepatitis C virus infection. Alcohol and other causes of liver diseases besides viral infections were found in 11.5% of the participants. A total of 90 (42.9%) participants had a history of alcohol consumption. Only 42 (20.0%) had a family history of liver disease. 

**Table 2:** Clinical Characteristics of Participants Data presented as proportion (N) and percentages (%)

Variables	Showing symptoms, n (%)	Not showing symptoms, n (%)
Orthopnoea	4 (1.9)	206 (98.1)
Paroxysmal nocturnal dyspnoea	4 (1.9)	206 (98.1)
Cough	24 (11.4)	186 (88.6)
Chest Pain	20 (9.5)	190 (90.5)
Palpitation	12 (5.7)	198(94.3)
Easy fatigability	96 (45.7)	114(54.3)
Bilateral Leg swelling	80 (38.1)	130 (61.9)
Abdominal swelling	120 (57.1)	90 (42.9)
Intermittent claudication	6 (2.9)	204 (97.1)
Hospital admission	110 (52.4)	100 (47.6)
Alcohol intake	90 (42.9)	120 (57.1)
Smoking of cigarettes	14 (6.7)	196 (93.3)
Hepatitis B viral infection	116(55.2)	94 (44.8)
Hepatitis C viral infection	70(33.3)	140 (66.7)
Family history of liver disease	42 (20.0)	168 (80.0)
Family history of heart disease	18 (8.6)	192 (91.4)

The mean pulse rate was 86.56±15.30 beats per minute and mean systolic and diastolic blood pressures were 116.90±17.28 mmHg and 77.09±11.30 mmHg, respectively (Table 3).

**Table 3:** Blood pressure and pulse rate of participants BP: blood pressure; bpm: beats per minute

Parameter	Minimum	Maximum	Mean ± SD
Pulse rate (bpm)	60	130	86.56±15.30
Systolic BP (mmHg)	70	170	116.90±17.28
Diastolic BP (mmHg)	40	100	77.09±11.30

**Figure 2:**
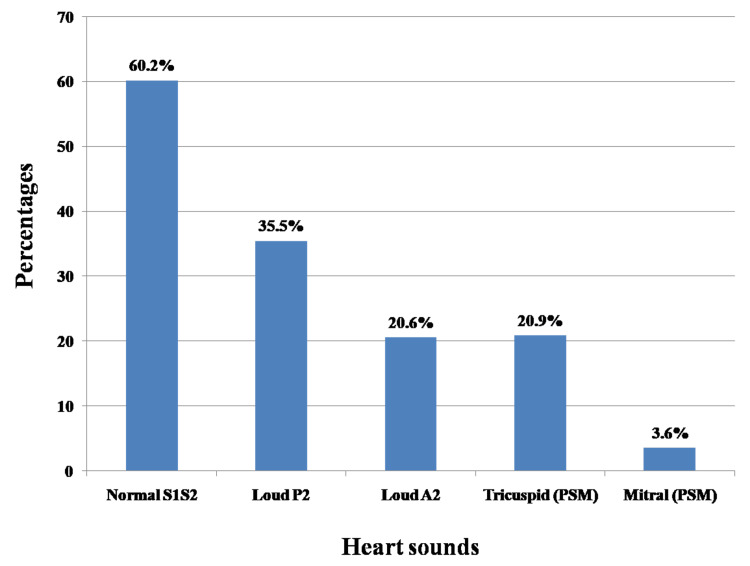
Heart sounds among study participants PSM: pan-systolic murmur

Normal heart sounds first and second were heard in only 126 (60.2%) participants (Figure 2).

Right ventricular systolic dysfunction

**Figure 3:**
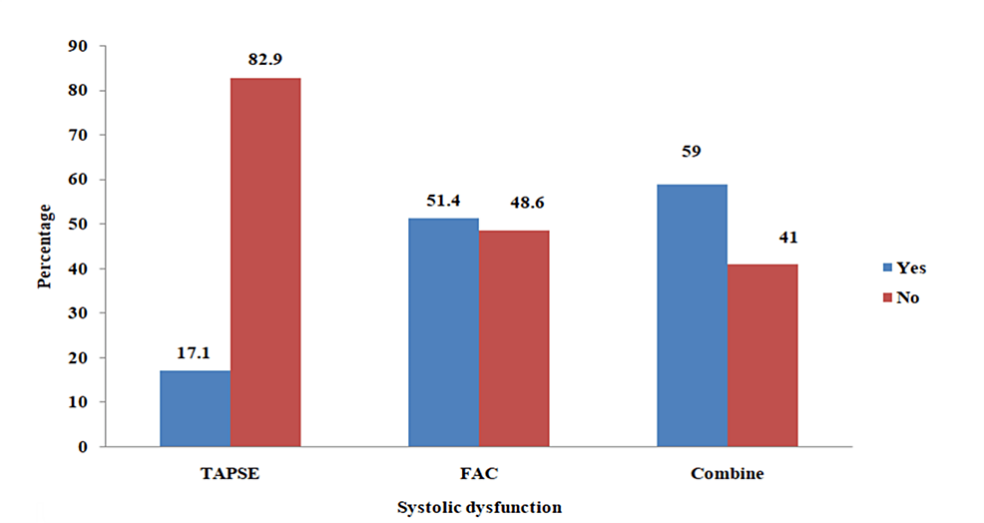
Prevalence of right ventricular systolic dysfunction in patients with liver cirrhosis Data presented as Percentage (%) Blue: Patients with systolic dysfunction; Red: Patients with normal systolic function TAPSE: Tricuspid Annular Plane Systolic Excursion; FAC: fractional area change

The right ventricular systolic function was assessed using TASPE and FAC. Using TAPSE, 36 (17.1%) participants had systolic dysfunction. Using FAC, 108 (51.4%) had systolic dysfunction. Adding systolic dysfunction using both TAPSE and FAC, 124 (59%) participants had right ventricular systolic dysfunction (Figure 3).

Right ventricular diastolic dysfunction

**Figure 4:**
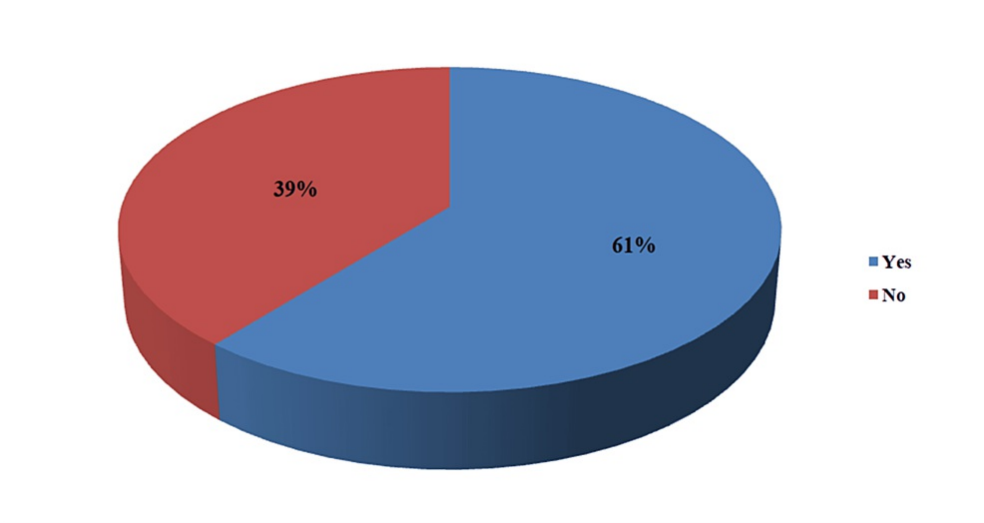
Prevalence of right ventricular diastolic dysfunction in patients with liver cirrhosis Data presented as Percentage (%)

**Figure 5:**
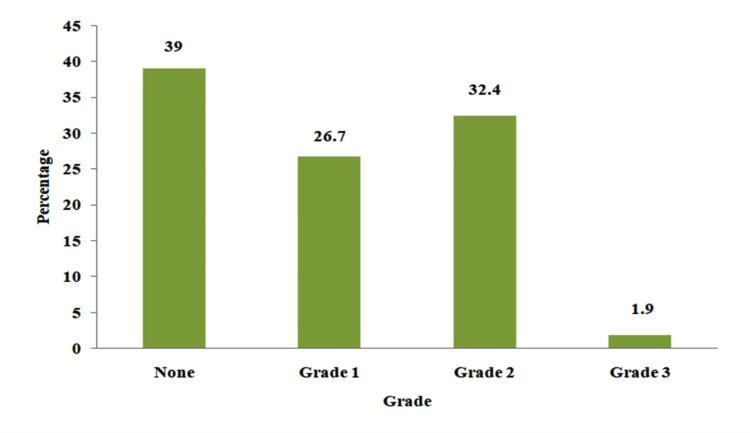
Prevalence of diastolic dysfunction and the various grades Data presented as Percentage (%)

Among the participants, 129 (61%) had right ventricular diastolic dysfunction (Figures 4, 5). Of these 129 participants, 56 (26.7%) had grade 1 diastolic dysfunction, 68 (32.4%) had grade 2 diastolic dysfunction, and four (1.9%) had grade 3 diastolic dysfunction.

Table 4 compares the FibroScan score of participants with systolic function. The result showed a statistically significant p-value comparing the FibroScans score of participants with systolic dysfunction using TAPSE and those with normal TAPSE (p-value = 0.002). There was no statistical significance comparing the FibroScan score of participants with systolic dysfunction using FAC and those with normal FAC values (p-value =0.614). There was no statistical significance comparing the FibroScan score of participants with diastolic dysfunction with those with normal diastolic function (p-value =0.590).

**Table 4:** Comparison of mean FibroScan scores and systolic and diastolic dysfunction Data presented as mean±SD P-values <0.05 is significant. The FibroScan scores of participants were compared across patients with systolic, and diastolic dysfunction versus those with normal function. Only TAPSE showed statistical significance. TAPSE: Tricuspid Annular Plane Systolic Excursion; FAC: fractional area change

	Mean ± SD	t-test	p-value
Systolic dysfunction (TAPSE)		3.163	0.002
Yes	40.25±3.92		
No	29.22±1.35		
Systolic dysfunction (FAC)		0.506	0.614
Yes	30.40±1.89		
No	31.75±1.88		
Systolic dysfunction (Combined)		0.808	0.420
Yes	31.94±1.77		
No	29.74±2.01		
Diastolic dysfunction		0.539	0.590
Yes	31.67±1.87		
No	30.22±1.85		

In Table 5, the FibroScan scores of participants were compared with different grades of diastolic dysfunction. Results showed that participants with grade 3 diastolic dysfunction had worse FibroScan scores which was statistically significant (p-value =0.018). The FibroScan score increases with the worsening of diastolic function.

**Table 5:** Comparison of mean FibroScans with grades of diastolic dysfunction Data presented as Mean±SD P-value <0.05 is significant

Grade	Mean ± SD	F-test	p-value
	30.22±1.85	3.445	0.018
Grade 1	25.41±2.71		
Grade 2	35.33±2.55		
Grade 3	44.40±4.97		

**Figure 6:**
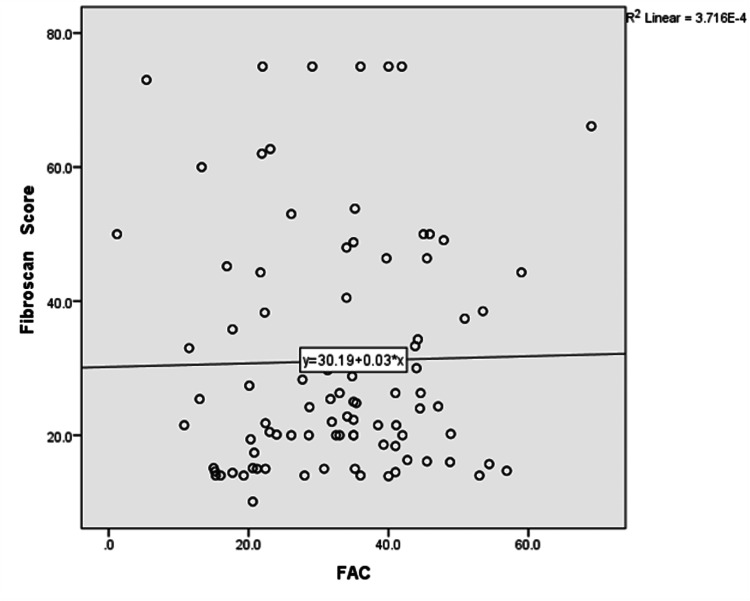
Scatter plot of FibroScan score against FAC Correlation coefficient (r) = 0.084;  p-value = 0.263 FAC: fractional area change

**Figure 7:**
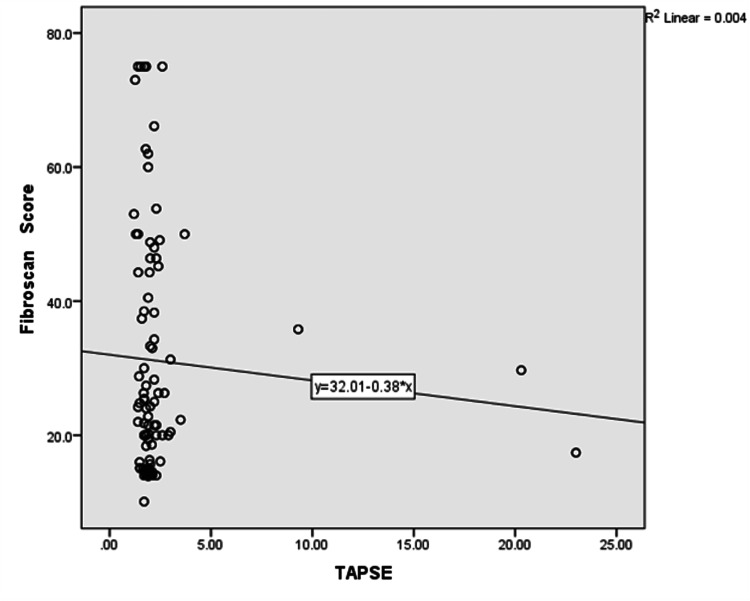
Scatter plot of FibroScan score against TAPSE Correlation coefficient (r) = -0.011, p-value = 0.882 TAPSE: Tricuspid Annular Plane Systolic Excursion

Figures 6, 7 are scatter plots of FibroScan score and FAC and TAPSE. There was no relationship between the FibroScan score and FAC. Still, there was a negative correlation coefficient when FibroScan scores were correlated with TAPSE indicating that participants with higher FibroScan scores had lower TAPSE value.

## Discussion

Two different methods were used to assess the right ventricular systolic function: TAPSE and FAC. In the evaluation of systolic function using TAPSE, 17.1% of the patients were found to have systolic dysfunction. Using FAC, 51.4% of the patients were found to have systolic dysfunction. There is an obvious difference between the results obtained from TAPSE and FAC. Assessment of right ventricular systolic function is quite challenging because of its unique geometry [25]. While cardiac magnetic resonance imaging is the gold standard for assessing right ventricular systolic function [25], a study has shown that FAC provides a more accurate estimate of right ventricular systolic function [26]. Various studies done to assess right ventricular systolic function among liver cirrhosis patients found impaired systolic function using right ventricular fractional shortening; right ventricular global strain and TAPSE did not show significant change compared with control [10]. This study did not use controls and thus, it is difficult to comment on the statistical significance of the above outcome.

Bekler et al. found systolic dysfunction using systolic tissue Doppler velocity of the tricuspid annulus S' among patients with liver cirrhosis across the various grades of hepatosteatosis [13]. Chen et al. reported no statistically significant difference in the systolic function among patients and control in their study which evaluated cardiac function in cirrhotic patients and its alteration with or without liver transplantation [17]. Most of the studies were comparative studies and thus did not report on the prevalence of systolic dysfunction. The difference observed in various studies might be due to the unique and complex anatomy of the right ventricle.

Diastolic dysfunction was found in 61.0% of patients. Among these participants, 26.7% had grade 1 diastolic dysfunction, 32.4% had grade 2 diastolic dysfunction and 1.9% had grade 3 diastolic dysfunction. Aziz et al. studied patients with liver cirrhosis and grouped participants into those with liver cirrhosis and hepatopulmonary syndrome, those with liver cirrhosis without hepatopulmonary syndrome, and controls [27]. They found diastolic dysfunction among all the participants with liver cirrhosis and hepatopulmonary syndrome; among participants with liver cirrhosis without hepatopulmonary syndrome, 72% had diastolic dysfunction, and 4% of controls [27]. The high prevalence of diastolic dysfunction is similar to the findings in this study. Also, a study done by Yasemin et al. showed significant right ventricular diastolic dysfunction in patients with liver cirrhosis [28].

Bekler et al. found an increase in the duration of IVRT in patients with liver cirrhosis [13]. Dinic-Radovic et al. found grade 1 diastolic dysfunction in 72.5% of the patients recruited, the DT was greater than 200 ms in 95% of their patients while the IVRT was greater than 80 ms in 75% of the patients [26]. Nasr et al. also observed grade 1 diastolic dysfunction of left ventricle in their study [29]. Bekler et al., in their study of right ventricular function in hepatosteatosis in non-alcoholic fatty liver disease (NAFLD), found no difference among patients and controls in E/A and DT, but found an increase in IVRT and that grades of hepatic steatosis positively correlated with right ventricular IVRT [13].

Chen et al. assessed only the diastolic function of the left ventricle and showed impaired diastolic function in 41.7% of patients [17]. Adebiyi et al. in their study of patients with liver cirrhosis on the left ventricle found an increase in the cardiac index but no significant statistical difference between the study group and control in their diastolic function [22]. Most studies have identified diastolic dysfunction in liver cirrhosis of both the right and left ventricles. The Nigerian study differs from others probably because of the small sample size and the method of assessing diastolic dysfunction used in the study. The study used only E/A, tissue Doppler, and other methods of assessing diastolic function not usually used by the researchers. Diastolic dysfunction has been found to have a worse prognosis than systolic dysfunction [22]. The difference noticed in the prevalence of diastolic dysfunction in various studies is probably because of different parameters used to assess diastolic dysfunction which include E/A, DT, IVRT, and E/e' by different researchers.

The current study is limited as two-dimensional echocardiography was used to carry out this study. A three-dimensional echocardiography would have provided an opportunity to do a volumetric study.

## Conclusions

Systolic and diastolic dysfunction were common among patients with liver cirrhosis. A high FibroScan score predicts worse diastolic dysfunction and the same with systolic dysfunction using TAPSE. Echocardiography should be made part of routine evaluation for patients with liver cirrhosis. This will help detect cardiovascular abnormalities like ventricular systolic and diastolic dysfunction and treatment instituted. Experimental studies should be carried out to find the impact of treatment on cardiovascular changes in patients with liver cirrhosis.
